# Defects of Nutrient Signaling and Autophagy in Neurodegeneration

**DOI:** 10.3389/fcell.2022.836196

**Published:** 2022-03-28

**Authors:** Jon Ondaro, Haizea Hernandez-Eguiazu, Maddi Garciandia-Arcelus, Raúl Loera-Valencia, Laura Rodriguez-Gómez, Andrés Jiménez-Zúñiga, Julen Goikolea, Patricia Rodriguez-Rodriguez, Javier Ruiz-Martinez, Fermín Moreno, Adolfo Lopez de Munain, Ian James Holt, Francisco Javier Gil-Bea, Gorka Gereñu

**Affiliations:** ^1^ Department of Neuroscience, Biodonostia Health Research Institute (IIS Biodonostia), San Sebastian, Spain; ^2^ Center for Biomedical Research of Neurodegenerative Diseases (CIBERNED), Madrid, Spain; ^3^ Department of Neurology, Care Sciences and Society, Center for Alzheimer Research, Karolinska Institutet (KI), Stockholm, Sweden; ^4^ Donostia University Hospital, San Sebastian, Spain; ^5^ Department of Clinical and Movement Neurosciences, Queen Square Institute of Neurology, Faculty of Brain Sciences, University College London, London, United Kingdom; ^6^ IKERBASQUE Basque Foundation for Science, Bilbao, Spain; ^7^ Department of Physiology, Faculty of Medicine and Nursing, University of Basque Country (UPV-EHU), Leioa, Spain

**Keywords:** neurodegenerative diseases, autophagy, nutrient sensing pathways, mitochondrial metabolism, lipid metabolism, glucose metabolism, mTORC1 (mechanistic target of rapamycin complex 1), AMPK

## Abstract

Neurons are post-mitotic cells that allocate huge amounts of energy to the synthesis of new organelles and molecules, neurotransmission and to the maintenance of redox homeostasis. In neurons, autophagy is not only crucial to ensure organelle renewal but it is also essential to balance nutritional needs through the mobilization of internal energy stores. A delicate crosstalk between the pathways that sense nutritional status of the cell and the autophagic processes to recycle organelles and macronutrients is fundamental to guarantee the proper functioning of the neuron in times of energy scarcity. This review provides a detailed overview of the pathways and processes involved in the balance of cellular energy mediated by autophagy, which when defective, precipitate the neurodegenerative cascade of Parkinson’s disease, frontotemporal dementia, amyotrophic lateral sclerosis or Alzheimer’s disease.

## Graphical Abstract

Schematic Representation Highlighting the Mechanisms of Nutrient Sensing and Autophagy Under Physiological Conditions.

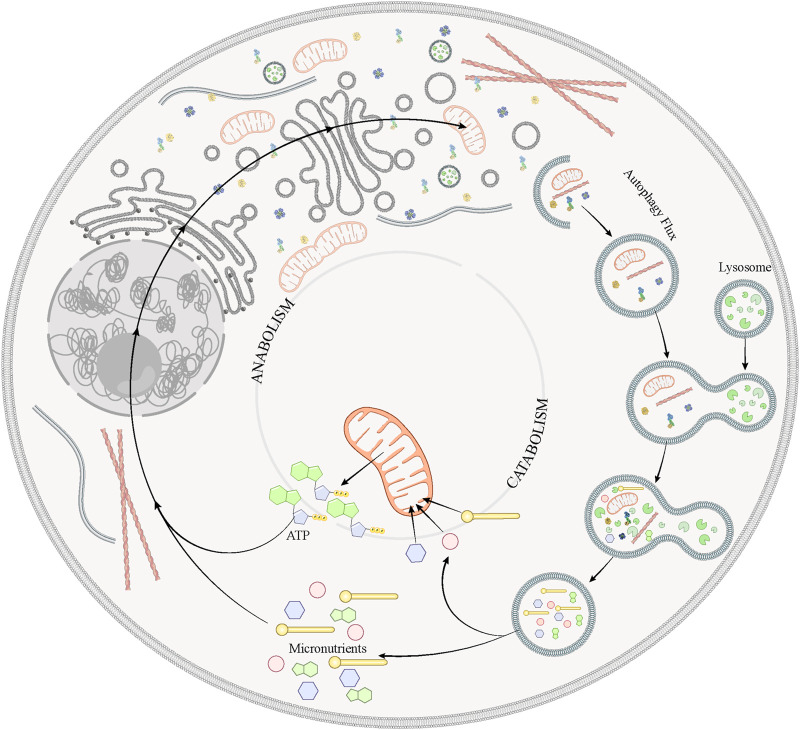

Under physiological conditions, the cell can sense changes in the availability of metabolic fuels and respond to these challenges through the reconfiguration of energy-producing pathways and recycling of accumulated or damaged organelles and proteins. For that purpose, the cell initiates the ALP-in which the damaged or unnecessary organelles and proteins are carried in the autophagosomes and degraded into the lysosomes-, generating micronutrients that will be used to obtain energy. The cell uses the energy produced and the micronutrients as building blocks to synthesize new macromolecules and organelles, which will respond to the cell requirements and maintain cell homeostasis.

## 1 The Importance of Autophagy to Access the Internal Nutrient Stores in Times of Energy Scarcity

Because the availability of environmental nutrients can be intermittent, cells and organisms have developed efficient ways to store nutrients during periods of abundance, as well as mechanisms to mobilize internal nutrient stores and reconfigure catabolic pathways to withstand periods of scarcity. Autophagy is the main cellular mechanism for the degradation and recycling of intracellular components, and thus of the utmost importance for adapting to and surviving nutrient limitation. This cellular process begins with the encapsulation of a cargo in a double membrane structure formed *de novo* called autophagosome, and culminates with the fusion of the lysosome and breakdown of the cargo into its basic building blocks, which will be used to produce energy or generate new cellular components. Thanks to pioneering studies in yeasts and later work in mammalian cells, we know that changes in the carbon source, as well as wholesale nutrient restriction, can activate macroautophagy (Mitchener et al., 1976). Multiple signaling mechanisms modulate autophagic activity in response to the nutritional status of the cell (Mehrpour et al., 2010), two of the most important of which are the mammalian target of rapamycin (mTOR) and the AMP activated protein kinase (AMPK).

MTOR comprises the mTOR 1 (mTORC1) and mTOR 2 (mTORC2) complexes. The mTORC1 complex is a nutrient sensing kinase that acts as an integrator of information on cellular energy balance and negatively regulates the initiation of macroautophagy. In the presence of nutrients and growth factors, mTORC1 is activated and supports anabolism and growth, while inhibiting catabolic pathways such as autophagy (Hosokawa et al., 2009) (Bar-Peled and Sabatini, 2014). Once activated, mTORC1 directly inactivates ULK1 (Unc-51 Like Autophagy Activting Kinase) by phoshorylation at Ser757 (Kim et al., 2011), a condition that is sufficient to inhibit autophagy in the presence of sufficient nutrients. Furthermore, mTORC1 can undirectly suppress autophagy by controlling lysosome biogenesis (Roczniak-Ferguson et al., 2012). mTORC1-mediated phosphorylation of the transcription factor EB (TFEB) decreases its transcriptional activity, thus decreasing the overall expression of autophagy-related gene expression (Roczniak-Ferguson et al., 2012). TFEB is the master transcriptional regulator of lysosomal and autophagy-related genes (Settembre et al., 2011).

he lysosome is the main mediator of cellular catabolism, being the key organelle in cellular degradation and recycling processes, which allows this organelle to senses cell’s nutritional status and give the initial response to the environmental changes. Thus, lysosome is at the center of a complex regulatory network for the control of cellular and organism homeostasis. The activation of mTORC1 requires its recruitment to the lysosomal surface, which is mediated by the amino acid-dependent activation of heterodimeric RAG GTPase and their interaction with Ragulator (Sancak et al., 2010; Lawrence et al., 2018; Lawrence and Zoncu, 2019). In this way, the information collected by the lysosome about the nutrient levels is transferred to mTORC1 to further regulate the biosynthesis pathways. The lysosome is also able to initiate the cellular response to a nutrient need through the activation of TFEB (Lysosomal mTORC1 and RAG GTPases modulate the nucleocytoplasmic shuttling of TFEB recruiting it in to the lysosomes) inducing the expression of genes that are related to the biogenesis of autophagosomes and lysosomes (Martina and Puertollano, 2013; Settembre et al., 2013). Recent findings have shown that lysosome is the place where AMPK can sense glucose availability as well in the absence of any changes in cellular energy state (i.e., AMP/ATP) through a mechanism that involves the formation of a complex with v-ATPase, ragulator, axin, liver kinase B1 (LKB1) acting in opposition to mTORC1 (Zhang et al., 2017).

AMPK is activated by nutrient limitation, energy deficiencies and other stress signals, and plays an essential role in the maintenance of metabolic homeostasis by activating key catabolic pathways while inhibiting anabolism. AMPK activation has classically been accepted to be triggerd by increased AMP/ATP ratios, but it was recently shown that it can be activated by glucose withdrawal even before AMP/ATP ratios increase (Zhang et al., 2017). AMPK promotes autophagosome formation through direct phosphorylation of ULK1 and ULK2 at Ser317 and Ser777 (Kim et al., 2011), and it can also indirectly activate autophagy by inhibiting mTOR through phosphorylation of tuberous sclerosis complex 2 (TSC2) and Raptor (Gwinn et al., 2008). Furthermore, AMPK is required during the TFEB-induced lysosomal biogenesis process; therefore, it is also capable of stimulating autophagic flux by increasing the number of lysosomal vesicles (Zhao et al., 2020). These studies have clearly demonstrated that AMPK, being a critical sensor of nutrient availability among other important cellular processes, is capable of coordinating autophagy activity through mTOR-dependent and independent mechanisms. Persistently high AMPK signaling as a consequence of energy shortages or dysregulated nutrient sensing would result in excessive autophagic flux and, consequently, in excessive elimination of certain organelles such as mitochondria, leading to further aggravation of the energy crisis. This is consistent with observations showing that persistent activation of the AMPK signaling pathway results in neuronal death *in vivo* (Ju et al., 2011; Domise et al., 2019; Belforte et al., 2021).

Although autophagy was originally described 60 years ago, numerous recent studies have reported autophagic alterations in different human disorders such as cancer and neurodegenerative diseases, among others (Mizushima et al., 2008; Mehrpour et al., 2010). Most of the studies that establish a connection between autophagy dysregulation and disease have emphasized the important role of autophagy in the maintenance of prosteostasis, in the quality control of intracellular organelles, its contribution to cell remodeling, or its role in innate and acquired immunity (Mizushima et al., 2008). In recent years, bioenergetic deficiencies combined with impaired autophagic responses to such challenges have emerged as novel potential drivers of disease, since they would ultimately aggravate the energy imbalance and compromise cellular homeostasis (Chakravorty et al., 2019). In highly specialized tissues with high-energy demands, the correct balance between energy production and utilization is essential to guarantee correct physiological functioning. The human brain consumes up to 20% of the body’s energy, predominantly directed towards biosynthesis, neurotransmission and defense against oxidative stress (Tefera et al., 2021). Therefore, during neurodegeneration or aging -both scenarios where energy-producing pathways are challenged and nutrients may be scarce-, the correct functioning of autophagy is not only necessary to recycle dysfunctional organelles and macromolecules, but it is also important for balancing nutrient needs and guaranteeing the availability of energy fuels, mainly in the form of glucose and fatty acids.

**FIGURE 1:**
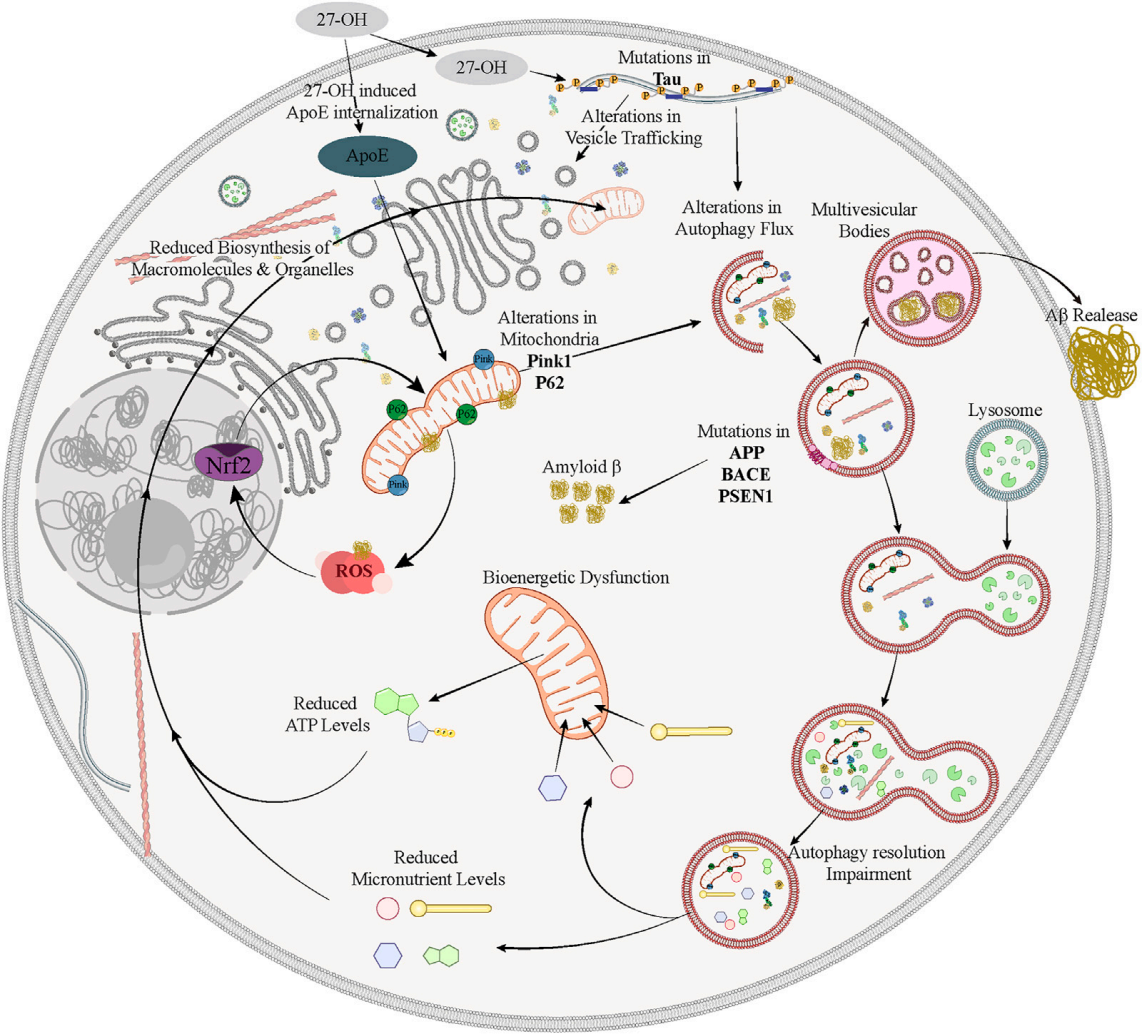
Genetic risk factors associated with poor metabolic fitness and defective autophagy in AD. In ApoE4 carriers, the overabundance of ApoE4 in brains threatens the proper lipid transport from astrocytes to neurons, thus leading to decrease autophagic biogenesis in neurons, poor mitochondrial recycling and, as a result, increased production of ROS, promoting pathological conditions that allow amyloid and Tau aggregation. Autophagosomes cointain the elements of the beta secretase, thus, mutations in APP, BACE1 and PS1 increase the production of Aβ, which further interferes with autophagosome formation and aggravates the oxidative stress status of the neurons. Besides, MAPT mutations increase the hyperphosphorylation of Tau and disrupt vesicle trafficking, impeding autophagosome formation and transport. On top of that, peripheral hypercholesterolemia may induces, through different ways, the accumulation of the brain cholesterol metabolite 27-OH, where it mediates a feed-forward loop that downregulates cholesterol synthesis in neurons and astrocytes. This will eventually cause detrimental effects including reduction of synaptic proteins (Psd95, SNAP-25) and Tau hyperphosphorylation, with consequences on autophagosome formation and trafficking, mitochondrial renewal and ROS production.

**FIGURE 2:**
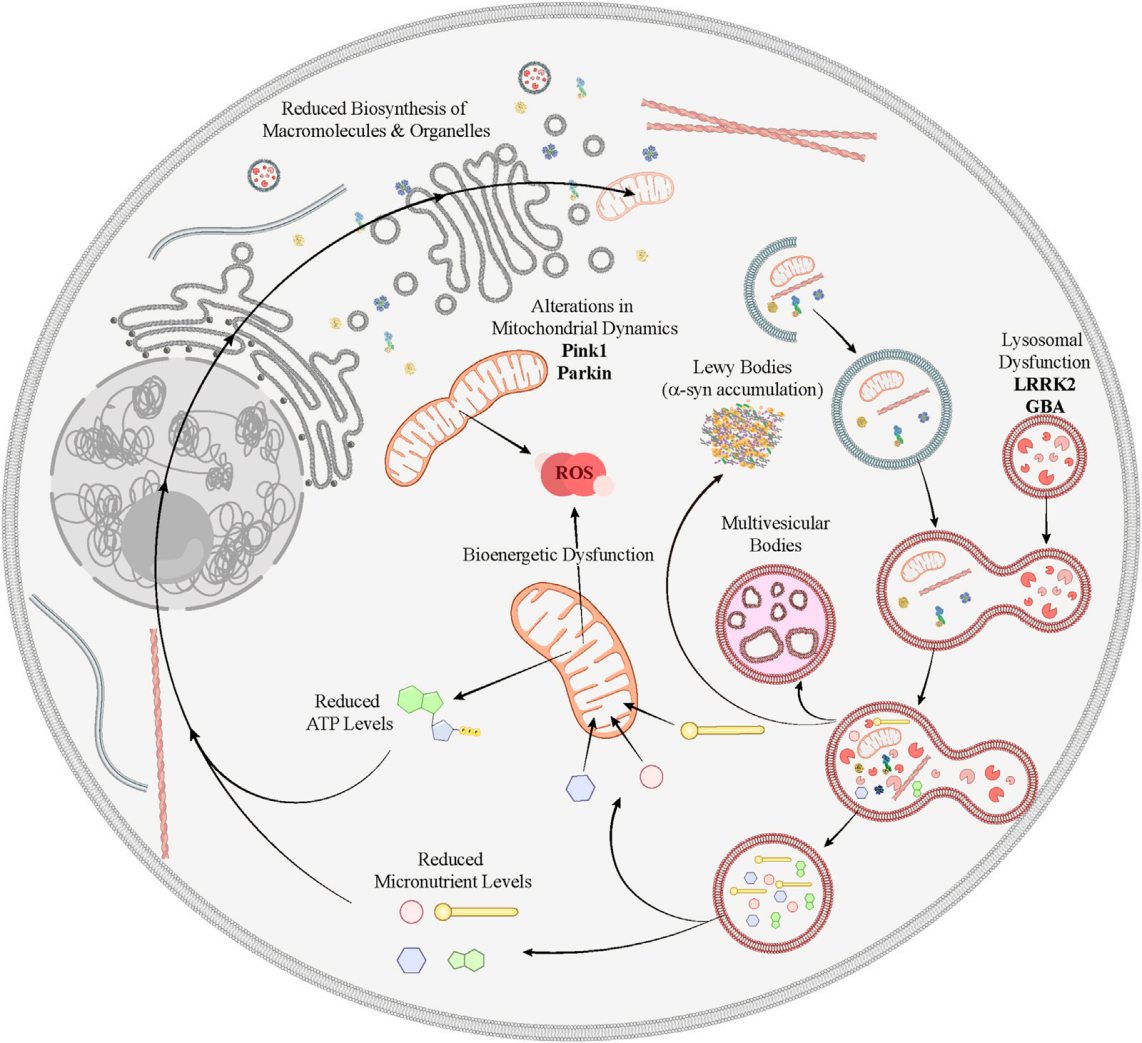
Schematic representation highlighting the involvement of various genetic risk factors associated to PD in defective mechanisms of nutrient sensing and autophagy. In PD, abnormalities in lysosomes caused by mutations in the genes LRRK2 or GBA would induce a collapse of the autophagic flux, leading to the accumulation of misfolded proteins into pathological inclusions, and intermediate autophagic vesicles, such as multivesicular bodies. Mutations in *PINK1* and *PARKIN* cause important alterations in mitochondrial dynamics, which affects severely the production of energy through the oxidative phosphorylation and increases the production of oxidative stress. This energy crisis, together with the failed attempt of the ALP to recycle micronutrients, jeopardize the biosynthesis of new macromolecules and organelles, and eventually compromise the neuronal homeostasis.

**FIGURE 3:**
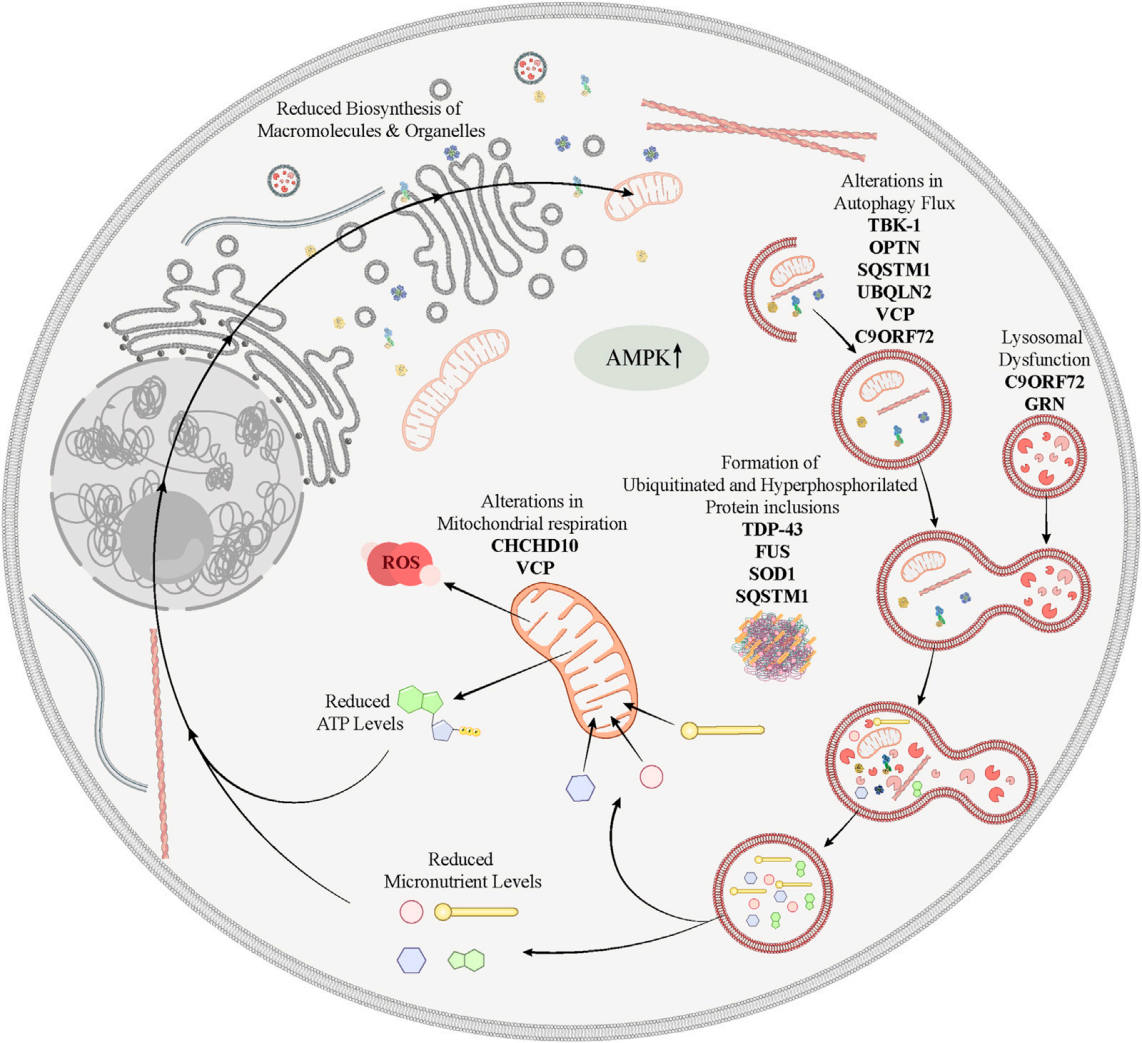
Schematic representation highlighting the involvement of various genetic risk factors associated to ALS/FTD in defective mechanisms of nutrient sensing and autophagy. In ALS/FTD, alterations in the autophagic flux caused by mutations in *TBK-1*, *OPTN*, *SQSTM1*, *UBQLN2*, *VCP*, *C9ORF72* or *GRN* may lead to the accumulation of misfolded proteins into pathological inclusions, and the improper recycling of damaged organelles, as for example mitochondria, to produce micronutrients. Damaged mitochondria or mutations in *CHCHD10* or *VCP* that are associated with defective mitochondria phenotypes, would further aggravate the energy crisis that is precipitated by the failure of energy-sensing pathways, such as defective lysosomal signaling or dysregulated AMPK activation.

Alterations in autophagy and in the pathways and processes of energy production or sensing are a fundamental part of the complex neuropathological cascade in many neurodegenerative diseases, and several of the mechanisms that connect both processes, i.e., AMPK and mTOR, have been widely studied as first-in-class targets to treat neurodegeneration. The following lines are intented to make a comprehensive review on the mechanisms involved in defective nutrient sensing and organelle reclycling that are common or specific to the most prevalent neurodegenerative diseases, and how risk factors, both environmental and genetic, impinge on them. The processes and mechanisms are further summarized in Table 1 and illustrated in Figures 1–3.

**TABLE 1:** Summary of altered mechanisms and related genes across neurodegenerative diseases.

	Risk factors related to nutrient dyshomeostasis	Altered cellular mechanism	mTOR/AMPK dysregulation	References
AD	Gene-related	—	Dysregulated mTOR response to starving	Lee et al. (2010)
	APOE4	Poor lipid utilization, Impaired lysosome and endosome sorting		
	PICALM		AMPK activation rescues amyloid pathology	Caccamo et al. (2010), Tramutola et al. (2017), Ou et al. (2018), Parcon et al. (2018)
	MAPT	Impaired autophagosome trafficking		
	Environment/lifestyle-related	—		Leoni et al. (2013), Vurusaner et al. (2014)
	Insulin resistance	Impaired brain glucose metabolism		
	Hypercholesterolemia	Impaired brain cholesterol metabolism		
PD	Gene-related	—	Upregulated mTOR activity	Kitada et al. (1998), Valente et al. (2004), Stefanis et al. (2001)
	Parkin, PINK1	Impaired mitochondrial recycling		
	SNCA, LRKK2	Impaired lysosome traffic and Impaired mitochondrial recycling		Ng et al. (2012), Zhu et al. (2019), Alegre-Abarrategui et al., 2009
	GBA	Defective lysosome activity and Mitochondrial dysfuncion		A et al. (2014), Magalhaes et al. (2016), Osellame et al. (2013); ME and AH. (2016)
	Environment/lifestyle-related	Impaired brain glucose metabolism		Chen et al. (2015), Cai et al. (2019)
	NA	—		
ALS/FTD	Gene-related	—	Chronic overactivation of AMPK signalling likely aggravates neuropathology	Bartolome et al. (2013), Straub et al. (2021), Lim et al. (2012a), Ling et al. (2014), Perera and Turner (2016)
	TARDBP, VCP, CHCHD10, SOD1	Impaired energy production in mitochondria		
		Impaired glucose uptake in MNs		
	C9ORF72, OPTN, SQSTM1, VCP, TBK1	Impaired lysosome biogenesis and maturation		Root et al. (2021), Sundaramoorthy et al. (2015), Deng et al. (2020), Ju et al. (2009)
	GRN (FTD)	link to metabolic disease and lysosome storage disorder		Youn et al. (2009), Smith et al. (2012)
	Environment/lifestyle-related (ALS)	—		
	Low BMI	—		O’Reilly et al. (2013), Perera and Turner, (2016)
	Type I diabetes mellitus	—		Jawaid et al. (2015)
	Strenuous sport exercising	—		Gallo et al. (2016), Julian et al. (2021)

NA, Not applicable.

## 2 Defects of Nutrient Signaling and Autophagy in Alzheimer’s Disease

Alzheimer’s Disease (AD) is the most prevalent age-related neurological disease worldwide, with its incidence increasing together with the rise of life expectancy. AD is characterized by the deposition of amyloid β protein (Aβ) in the form of extracellular amyloid plaques and by the presence of intracellular Neurofibrilary tangles (NFT) formed by hyperphosporylated Tau protein aggregates (TAU) (De Strooper and Karran, 2016). Aβ is genrated by the cleavage of APP by β-secretases (BACE1) and by Presenilin 1 and 2 (PS1 and PS2). Mutations in APP, PS1, PS2 or BACE1 (Reitz et al., 2020) have been shown to be responsible for the familiar cases of the disease, that show an early onset (EOAD). However, the vast majority of AD cases (95%) are sporadic, present a late onset (LOAD) and the causes remain largely unknown, although its thought to be due to a combination of environmental and genetic risk factors, in addition to lifestyle. Apolipoprotein E4 (ApoE4) allele is the most significant genetic risk factor for LOAD, being present in more than 50% of the cases (Raber et al., 2004). The main role of ApoE is cholesterol transport, but it is also involved in Aβ processing and glucose metabolism (Jiang et al., 2008). Indeed, regional glucose hypometabolism is an eary event in the pathogenesis of the disease, being used even for diagnosis purposes, and ApoE4 carriers show decreased glucose metabolism compared to non-carriers (Ong et al., 2014; Johnson et al., 2017).

Considering that glucose is a major source of energy in the brain, it is possible that in the context of AD, when deficiencies in glucose metabolism start to occur, autophagy plays a key role in order to acces internal sources of nutrients to maintain the bioenergetic homeostasis. However, deregulation of nutrient-sensing pathways and defects in autophagy and in the clearance of dysfunctional proteins have also been linked to AD pathophysiology (Nilsson and Saido, 2014; Hou et al., 2019). Therefore, a better understanding of pathways such as the regulation of mTORC1 and AMPK-induced activation of autophagy may offer novel potential treatments for age-related diseases.

### 2.1 Evidence of Poor Nutrient Sensing in Alzheimer’s Disease

A seminal work on the role of autophagy in AD identified a higher accumulation of autophagosomes in the frontoparietal cortex of affected patients through immuno-electron microscopy (Nixon et al., 2005). Later, another study showed Beclin-1 to be significantly reduced in AD brains (Pickford et al., 2008). Upregulation of microtubule-associated light chain 3-ll (LC3-ll), which induces autophagy, is seen at early AD stages (Yu et al., 2005). Moreover, genes that regulate autophagy, such as the phosphatidylinositol-binding clathrin assembly protein (PICALM), are correlated with the accumulation of tau in zebrafish and Drosophila models (Moreau et al., 2014) and have emerged as genetic risk factors for AD (Lee et al., 2011; Ortega-Rojas et al., 2016; Santos-Rebouças et al., 2017). Indeed, PICALM cleavage is altered in AD (Carrasquillo et al., 2010; Schnetz-Boutaud et al., 2012), leading to reduced levels that result in a lack of autophagosome maturation of (Lee et al., 2010). Also, autophagy is disrupted by PS1 mutations related to EOAD (Lee et al., 2010). Decreased lysosomal acidification, a cause of autophagy dysfunction, has been observed in fibroblasts from EOAD PS1 A246E mutation carriers and it’s linked to mutations in a vacuolar ATPase (v-ATPase), responsible for pumping protons into the lysosome (Coffey et al., 2014). In PS1 deficient cells, the amino-acid sensing mTORC1 is attached to lysosomal membranes and does not react to starvation, restricting lysosomal function (Reddy et al., 2016). These findings suggest that nutrient-sensing pathways could play a central role in the pathogenesis of AD.

mTORC1 is one of the most studied amino acid nutrient sensing signaling pathwys and plays a key role in both Aβ and TAU pathologies. Aβ accumulation has been associated to the overactivation of mTORC1 signaling, whilst mTOR signaling inhibition leads to reduced Aβ levels (Caccamo et al., 2010; Kioussis et al., 2021). Moreover, accumulating evidence suggests that mTOR activation enhances TAU pathology via autophagy inhibition which, in turn, enhances Aβ accumulation (Tramutola et al., 2017), that further promotes TAU hyperphosphorylation. In both cases, mTORC1 inhibition seems to be beneficial to diminish Aβ and TAU pathology (Wang et al., 2014), although mTORC1 regulation is complex and needs to be better understood in order to design effective treatments with minimal side-effects (Carosi and Sargeant, 2019).

Together with mTORC1, AMPK regulates glucose and lipid metabolism, it is deregulated in AD brains (Jeon, 2016). Numerous reports show that AMPK signalling modulates TAU phosphorylation and pathology *in vivo* (Domise et al., 2016; Wang et al., 2020b, 2020a). Further, AMPK is an autophagy activator, thus its activation should be beneficial to reduce Aβ pathology. For example, treatment with metformin, a known AMPK activator, induced a decrease of Aβ pathology in an *in vivo* model of AD (Ou et al., 2018). Also, some studies have shown that metformin is associated with a lower risk of AD (Ng et al., 2014; Chin-Hsiao, 2019). However, this issue remains controversial, as at least one other study found no correlation between metformin and AD risk in diabetic patients (Sluggett et al., 2020), and other studies have found an increased risk of AD with metformin intake (Imfeld et al., 2012; Ha et al., 2021). The discrepancies between these reports might be explained by inter-individual differences in the pathogenesis of AD and highlight the relevance of identifying AD subtypes for personalized treatments (Loera-Valencia et al., 2019a; Caberlotto et al., 2019). Altogether, it becomes evident that nutrient metabolism and autophagy are crucial regulators of pathways with relevant roles in TAU and Aβ aggregation (Tang et al., 2014; Yang et al., 2019).

### 2.2 Autophagy Dysregulation in the Context of Alzheimer’s Disease: Focus on Disease-Relevant Genes and Metabolic Risk Factors

#### 2.2.1 Amyloid β Protein

The relationship between autophagy and amyloidosis is complex since many features of proteolytic cleavage are involved in both the clearance and production of amyloid-beta. Inhibition of autophagy with chloroquine (CQ) in SH-SY5Y cells and the HEK293T (AβPPsw) model induces intracellular accumulation of Aβ1-42 (Gerenu et al., 2021), while autophagic activation by estrogen receptor beta overexpression, decreased Aβ1-42 levels (Wei et al., 2019). However, Nilsson and co-workers previously reported that a conditional knockout of Atg7 (with impaired autophagy), decreased amyloid-beta secretion by 90%, and normal Aβ secretion was restored when autophagy was stimulated to normal levels (Nilsson et al., 2013; Loera-Valencia et al., 2019b). This is evidence that autophagy is important not only for Aβ degradation but also for its production, this theory is supported also by the presence of the four components of the beta-secretase complex in autophagic vesicles (Yu et al., 2005).

Although the exact mechanism regulating the formation of the isolation membrane that leads to autophagosome formation is not fully understood, it has been reported that mitochondria-endoplasmic reticulum (ER) contact sites (MERCS) are one of the places where this process can occur (Garofalo et al., 2016). MERCS are domains arising from the interaction between specific regions of the ER and the outer mitochondrial membrane. These contact sites have been related to important biological processes such as the ER calcium shuttling into mitochondria and they are altered in AD and in AD-related models (Paillusson et al., 2016). Moroever, all required components for APP processing as well as Aβ peptide can also be found in MERCS (Schreiner et al., 2015). It was recently shown that the connectivity between mitochondria and ER is increased in brains and primary neurons isolated from AD mouse models displaying increased amyloidosis. In addition to MERCS ultrastructural alterations, neurons from AD mouse models also displayed alterations in autophagosome formation, mitochondrial membrane potential and ATP production during starvation (Leal et al., 2020).

#### 2.2.2 MAPT (Tau Protein Aggregates)

Autophagy is also related to the clearance of TAU as an alternative pathway for degradation by the ubiquitin-proteasome system. Swedish amyloid precursor protein gene double-mutation KM670/671NL (APPswe) EOAD patients show hyperphosphorylated tau associated with LC3 and p62 (a ubiquitin-binding protein) in their frontal cortex, which is not present in control subjects (Piras et al., 2016). Again, the conditional knockout model of Atg7 shows impaired autophagy, age-related neurodegeneration, and accumulation of hyperphosphorylated tau in the brain (Inoue et al., 2012). Reciprocally, pathogenic tau mutations such as the tubulin-binding repeats linked to chromosome 17 can induce accumulation of acid lysosomes and interfere with normal autophagic flux (Lim et al., 2001). Surmounting evidence shows the importance of autophagy for AD and other neurodegenerative diseases; nevertheless, the classic view of autophagy as a recycling system of the cell is becoming outdated, since it can actively modulate mechanisms of synaptogenesis and cell death (Nikoletopoulou et al., 2015; Jung et al., 2020). Therefore, we need to study these new roles if a feasible autophagy-based AD therapy is to be developed.

#### 2.2.3 ApoE4

As we mentioned before, ApoE is a lipoprotein that functions as a transporter of cholesterol and other lipids in the body (Holtzman et al., 2012). ApoE has three isoforms (ε2, ε3 and ε4) where ApoE4 confers a higher risk to develop AD and is regarded as the strongest genetic risk factor for LOAD (Kloske and Wilcock, 2020).

Several studies point to ApoE4 involvement in autophagy dysfunction. FoxO3a, a member of a family of transcription factors that play a role in the regulation of several autophagy-related genes (Audesse et al., 2019), is repressed in human postmortem brain samples from AD ApoE4 carriers in comparison to non-carriers (Sohn et al., 2021). This study also showed a decrease in protein expression of autophagy- and mitophagy-related genes, such as ATG12, BECLIN-1, BNIP3 and PINK1 that are regulated by FoxO3a. Another study reported that the coordinated lysosomal expression and regulation (CLEAR) DNA motifs, a region in the DNA where the master autophagy regulator and TFEB induces its downstream genes, can also be targeted by ApoE4, which competitively binds CLEAR motifs, inhibiting the expression of autophagy-related genes (Parcon et al., 2018).

Cell-type-specific studies show that astrocyte metabolism is affected by ApoE4 expression (Figure 1). Upon autophagy-inducing conditions, ApoE4 expressing astrocytes exhibited lower autophagic flux in comparison to ApoE3 astrocytes (Simonovitch et al., 2016). Moreover, APOE4 expression was associated with altered mitochondrial dynamics including, fusion, fission, and mitophagy in comparison to ApoE3-expressing astrocytes (Schmukler et al., 2020). A recent study investigated the role of ApoE4 in regulating fatty-acid metabolism in the brain (Qi et al., 2021) and found that ApoE4 astrocytes had more fragmented mitochondria, lower β-oxidation levels and a higher accumulation of lipid droplets. These effects could in turn contribute to the lipid dysregulation and bioenergetic deficits that are observed in AD (Sato and Morishita, 2015).

### 2.3 Lipid and Cholesterol Sensing in Alzheimer’s Disease

#### 2.3.1 Alterations in Cholesterol Metabolism Lead to Inflammation and Synaptic Dysfunction in the Brain

Cholesterol nuclear receptors are widespread in neurons and glial cells alike (Olkkonen and Levine, 2004; Ali et al., 2013; Moutinho et al., 2015). Cholesterol levels in the brain are regulated by the interplay between oxidized forms 24-hydroxycholesterol (24-OH) and 27-hydroxycholesterol (27-OH) (Meaney et al., 2007; Båvner et al., 2010; Shafaati et al., 2011; Ali et al., 2013). As cholesterol is metabolized by the enzyme CYP46A1 into 24-OH in neurons, HMG-CoA reductase is activated to promote cholesterol biosynthesis in the brain. Conversely, high levels of 27-OH coming from peripheral cholesterol catabolism decrease HMG-CoA reductase activity and decrease global cholesterol levels in the brain. Neurodegeneration depletes the brain from CYP46A1 and thus decreases cholesterol biosynthesis (Leoni et al., 2013), which is essential for memory function. Similarly, hypercholesterolemia leads to the accumulation of 27-OH in the brain, which disrupts cholesterol metabolism as discussed below.

Cholesterol in the brain is synthesized *de novo* by glial cells, transporting it to neurons via lipoprotein particles, such as APOE (Figure 1). When cholesterol synthesis disruption causes neuronal function impairment, showing behavioral defects in mice (Ferris et al., 2017). Cholesterol metabolism deregulation can alter astrocytes and microglia physiological states. High levels of 27-OH generated from a high-fat/cholesterol diet led to overproduction of S100A8 alarmin and its receptor RAGE, inducing sterile inflammation that contributes neurodegeneration (Loera-Valencia et al., 2021a).

Besides pro-inflammatory responses, 27-OH is also able to activate cellular pathways that are regulated by reactive oxygen species (ROS) such as autophagy (Vurusaner et al., 2014). By activating the extracellular signal-regulated kinase (ERK) and the phosphoinositide 3-kinase (PI3K)/Akt pathways, 27-OH induces the upregulation of the nuclear factor erythroid 2 p45-related factor 2 (Nrf2) *in vitro*. Nrf2 is a transcription factor for several antioxidant proteins that plays a role modulating autophagy via p62 in response to oxidative stress (Tang et al., 2019). *In vitro* treatments of 27-OH induced an increase in Beclin1, LC3II and a decrease in p62 (Vurusaner et al., 2018).

Cholesterol metabolism alterations can also directly impact synaptic function (Figure 1). Evidence shows that APOE4 has low transport affinity and binding capacity for lipids reducing cholesterol transport from astrocytes to neurons and leading to lower synaptic density and cognitive decline (Lee et al., 2021). Cholesterol imbalance in the brain can worse memory performance in mice (Merino-Serrais et al., 2019), lead to synaptic dysfunction by downregulating Psd95 and SNAP-25 (Merino-Serrais et al., 2019).

#### 2.3.2 CD36 and Lipid Sensing in the Brain

While neuroendocrine neurons sense fatty acids to modulate metabolism in the body (Oishi et al., 1990; Tewari et al., 2000; Honen et al., 2003), at the cellular level, the molecular sensor for fatty acids in brain cells is CD36, a transmembrane glycoprotein that is expressed in many cell types, including epithelial cells, adipocytes, dendritic cells and hepatocytes, among others (Mitchell et al., 2011). Although CD36 plays a role in the binding, transport and uptake of fatty acids (Pepino et al., 2014; Ioghen et al., 2021) as well as fatty-acid sensing in oral epithelial cells (Laugerette et al., 2005), it has multiple cellular functions such as angiogenesis or internalization of bacteria depending on the cell type where it is expressed on (Abumrad et al., 2005).

Early studies on Alzheimer’s Disease patients showed that CD36 is highly expressed in cortical samples of AD patients and cognitively normal subjects with the presence of amyloid plaques compared with amyloid-free controls (Ricciarelli et al., 2004). This study pointed at an association between CD36 and Aβ in human brains independently of AD occurrence. However, a genetic study of 859 AD patients revealed that a polymorphism in the CD36 gene significantly associated the risk for developing AD (Šerý et al., 2017). In the early stages of AD, activated microglia and macrophages induce the expression of CD36 in response to Aβ (Ricciarelli et al., 2004). The interaction between Aβ and CD36 has been shown to activate the NLRP3-inflammasome system, inducing the production of pro-inflammatory cytokines (Sheedy et al., 2013) and ROS (Coraci et al., 2002; Bamberger et al., 2003). It is also interesting to consider the possibility that early autophagy induction by CD36 in response to Aβ can also increase the autophagy-mediated secretion of oligomers from multi-vesicular bodies in the brain, thus contributing to seeding and protein aggregation (Figure 1) (Nilsson et al., 2013; Loera-Valencia et al., 2019b).

## 3 Defects of Nutrient Signaling and Autophagy in Parkinson’s Disease

Parkinson’s disease (PD) is a common neurodegenerative disorder that currently affects 1% of people over 65 years of age (Aarsland et al., 2021), and whose prevalence is expected to double by 2060 in the United States (Savica et al., 2018). As well as bradykinesia, rigidity or rest tremor (Postuma et al., 2015), PD often features non-motor symptoms (NMS), such as cognitive impairment or sensory alterations (Chaudhuri and Schapira, 2009). Pathophysiologically, PD is characterized chiefly by a slow and progressive degeneration of dopaminergic neurons in the Substantia Nigra (Drui et al., 2014), which is the cause of most of the motor symptoms, although non-dopaminergic neurons are also affected in PD (Schneider and Alcalay, 2017; Stoker and Greenland, 2018). While the underlying causes of the neurodegenerative process in PD are not fully understood, it evidently stems from a combination of cell-autonomous and non-cell-autonomous mechanisms. The former include, altered mitochondrial bioenergetics, impaired protein recycling (Al-Bari and Xu, 2020), where the master regulators mTOR and AMPK are crucial nutrient sensors (Hardie et al., 2012; Tan and Miyamoto, 2016), whereas protein aggregation and neuroinflammation are prominent non-cell-autonomous contributors to PD pathology, and it is believed to pathogenesis (Poewe et al., 2017). The development of dopaminergic neurons is an energy demanding process owing to the extensive branching of dendrites. This high-energy demand suggests nutrient and energy sensing, *via* mTOR and AMPK could be key disease development modifiers. In fact, much of the future work investigating defective organelles and metabolic abnormalities in PD is expected to involve analysis of specific proteins implicated in PD *via* pathological and genetic studies. Hence, here we focus on the effects of PD causing gene mutations on energy balance and the autophagolysosomal pathway, and their relationship to nutrient-sensing abnormalities.

### 3.1 Evidence of Poor Nutrient Utilization and Autophagy Dysregulation in Parkinson’s Disease Patients

PD patients are neuropathologically characterized by α-synuclein (α-syn) Lewy Bodies (LBs) neuronal inclusions, not efficiently metabolized by abnormal autophagy observed in dopaminergic neurons in the substantia nigra in PD brains (Anglade et al., 1997). Phosphorylated ubiquitin, as mitophagy tag, is increased in PD patients and correlates with tau tangles and levels of Lewy Bodies (LB) (Fiesel et al., 2015; Hou et al., 2018). Moreover, LC3II has been localized in LBs and its levels were significantly increased in PD samples (Dehay et al., 2010). In contrast, lysosomal enzymes activity is impaired, showing that autophagy is an important cellular process in PD pathophysiology.

Recycling of cell components is most important during starvation and so nutrient sensing and energy producing and regulating systems should always be considered in conjunction with autophagy. Increased expression of mTOR has been detected in the temporal cortex of PD patients with aggregations of α-syn (Crews et al., 2010; Zhu et al., 2019). Moreover, the inhibition of autophagy that accompanies elevated α-syn expression is attributed to mTOR activation (Gao et al., 2015; Zhu et al., 2019). Equally, in cultured cells and neurons with mutant α-syn mTOR signaling is upregulated and autophagy repressed, with the latter being reversed by mTOR inactivation (Jiang et al., 2013; Zhu et al., 2019). Although mTOR inactivation restores autophagy in PD cell models, it is crucial to avoid complete inhibition, because mTOR is essential for neuronal growth and survival and it regulates many important processes, such as synaptic plasticity and memory formation (Bekinschtein et al., 2007). Therefore, a balance between induction of autophagy and a basal mTOR activity has to be achieved for optimal brain function (Zhu et al., 2019).

AMPK plays a major role in regulating energy metabolism, via activation of catabolism and repression of energy-consuming processes (Hang et al., 2015), and AMPK activation can be neuroprotective during glucose starvation (Culmsee et al., 2001). AMPK appears to impact dopaminergic neuronal homeostasis in conjunction with the protein Parkin, is involved in recycling mitochondria (Zong et al., 2002). Concordantly, AMPK activation by metformin ameliorates the locomotion defects of Parkin-null flies, whereas this protective effect disappears when AMPK is silenced. Overexpression of AMPK also partially rescued the mitochondrial abnormalities of Drosophila that express mutated human leucine rich repeat kinase 2 (LRRK2), LRRK2^G2019S^ (Ng et al., 2012). Furthermore, resveratrol, a strong activator of AMPK in neurons, increased mitochondrial biogenesis and autophagic flux in fibroblasts carrying defective Parkin (Ferretta et al., 2014). Conversely, prolonged activation of Parkin (Van Rompuy et al., 2014) or AMPK have been shown to be detrimental for dopaminergic neurons, so both Parkin and AMPK can be neuroprotective or neurotoxic depending on the context, although the reasons behind these diverse effects are not known (Hang et al., 2015). Therefore, further research is needed, to determine the degree and duration of the Parkin-AMPK activation that achieves positive effects on neuron function and survival (Hang et al., 2015).

Autophagolysosomal and mitochondrial function are integrated with lipid metabolism and there are multiple lines of evidence indicating that lipid homeostasis is perturbed in PD. For example, (Zambon et al., 2019), showed a marked reduction in cholesterol levels in dopaminergic neurons of PD patients derived from iPSCs, together with reduced expression of CYP46A1, an ER enzyme crucial for cholesterol metabolism (Liu et al., 2010; Zambon et al., 2019). Moreover, some studies have linked α-syn toxicity with lipid droplet accumulation in human iPSC-derived neurons, (Fanning et al., 2019), whereas an, ultrastructural analysis of LB of PD brain found accumulated lipid vesicles, membrane fragments and cytoskeletal elements (Shahmoradian et al., 2019). Furthermore, lipid accumulation has been implicated in several events associated with PD, such as lysosomal blockade and neuroinflammation (Hallett et al., 2019). Hence, lipid metabolism is a highly promising target for PD.

Although altered cell metabolism is involved in PD pathogenesis, further research is needed to elucidate the fundamental basis of the metabolic alterations, as well as to understand better the network of interactions that encompasses the diverse mechanisms implicated in PD described above. The improved knowledge is expected to clarify and reconcile, or refute, the following contradictory results. While some studies reported a reduced risk of PD in patients with Type 2 diabetes (T2D) (Sääksjärvi et al., 2015), others find the opposite (Hu et al., 2007; Sun et al., 2012), or no relation between them (Palacios et al., 2011). Similar inconsistencies have been described in relation to cholesterol metabolism, indicating that modifying factors could be modulating the association between blood cholesterol and PD risk (de Lau et al., 2006; Mascitelli et al., 2009).

### 3.2 Evidences of Mitochondrial Dysfunction in Parkinson’s Disease

Mitochondria have been heavily implicated in PD since the landmark finding that PD brains have low respiratory complex I activity (Schapira et al., 1989) and that the complex I inhibitor rotenone induces dopaminergic cell death in rats (Alam and Schmidt, 2002). Although the entire respiratory chain depends on proteins encoded in mitochondrial DNA (mtDNA), defects in mtDNA can predominantly affect complex I (Dunbar et al., 1996). Moreover, mutant mtDNAs accumulate with age, and more so in PD patients (Bender et al., 2006), or alternatively PD patients display low mtDNA numbers (Tzoulis et al., 2013). Mitochondrial DNA is further implicated in the PD developmental cascade by the fact that a variety of defective nuclear genes that adversely impact mtDNA cause parkinsonism, featuring DPN cell death and levodopa responsive motor dysfunction (Miguel et al., 2014; Pedroso et al., 2018).

Further interest in mitochondrial involvement in PD was sparked by the findings that mutations in key factors required for mitochondrial recycling, Parkin and PINK1, cause familial PD (Kitada et al., 1998; Valente et al., 2004). These findings were all the more provocative as other recycling factors were implicated in the disease, and defective autophagy is a recurring theme in other neurodegenerative disorders (Menzies et al., 2015; Guo et al., 2018). Moreover, as impaired autophagy can result from mitochondrial dysfunction, and respiratory complex I is needed for maximal autophagy (Thomas et al., 2018), the activities of mitochondria, autophagosomes and lysosomes are coupled; hence, problems with these organelles are concordant, rather than competing, explanations for the development of PD.

Changes in mitochondrial function in PD could stem from, or be exacerbated by, altered nutrient metabolism given that the brain requires a continuous supply of energy in the form of ATP, much of which is produced from glucose, either *via* glycolysis or additionally by its further oxidation in mitochondria (Cunnane et al., 2020). In fact, decreased glucose metabolism have been observed in PD patients (Eberling et al., 1994; Ahmed et al., 2009). Interestingly, glucose deprivation promotes α-syn aggregation (Bellucci et al., 2008), and impaired bioenergetics and reduced ATP levels might contribute to the pathogenesis of PD (Cai et al., 2019). Furthermore, Cai and colleagues suggested that glycolysis could be a new therapeutic target for PD, as Terazosin, a drug used to treat prostatic hyperplasia and hypertension, has neuroprotective effects in multiple PD models. Terazosin, in addition to blocking α-adrenergic receptors, enhances glycolysis by stimulating phosphoglycerate kinase 1 (PGK1) activity; and consequently, increases oxidative phosphorylation and thus ATP levels (Chen et al., 2015). This enhancement of PGK1 activity increases dopamine levels, slows or prevents neurodegeneration and improves motor performance in several animal models of PD, such as the MPTP mouse, OHDA rat, and PINK1 and LRRK2 fly models (Cai et al., 2019).

### 3.3 Parkinson’s Disease Causing Genes as Drivers of Metabolic Alteration

Although a large majority, 85–90%, of PD cases are sporadic there is intense interest in the monogenic causes of PD, as they can potentially reveal the underlying processes that are central to all forms of PD. Among the cellular mechanisms of the mutant proteins that cause familial PD are ones related to protein folding and aggregation, and phosphorylation of α-syn (encoded by the SNCA gene), neuroinflammation, intracellular vesicular trafficking, lysosomal and mitochondrial function (Hong et al., 2011; Hirsch et al., 2013; Ryan et al., 2015; Poewe et al., 2017; Kuhlmann and Milnerwood, 2020). The genetic study of PD started in 1997 with the discovery of a missense variant (A53T) in *SNCA* in a large Italian family with PD (Polymeropoulos et al., 1996). Currently, more than 20 genes have been identified as PD causing genes (SNCA, PARKIN, PINK1, DJ1, LRRK2, GBA, VSP35…) (Blauwendraat et al., 2020). Here, we focus on three genes with strong links to nutrient-sensing, energy metabolism and recycling pathways (Figure 2).

SNCA. α-syn protein is a natively soluble protein encoded by SNCA gene. Under non-pathological conditions, α-syn is predominantly localized in the presynaptic terminal, where it plays important roles in transmembrane transport, intracellular trafficking and cell recycling pathways, including autophagy, Chaperone-Mediated Autophagy (CMA) or proteasome pathways (Seidel et al., 2010; Hong et al., 2011; Cannon et al., 2013; Eisbach and Outeiro, 2013; Oaks et al., 2013; Blauwendraat et al., 2020). In PD, abnormally aggregated α-syn is the major component of LBs (Iwatsubo, 2003).

Study of the α-syn mutant, A53T, (A53T) has revealed alterations in SIRT1, a NAD^+^-dependent deacetylase involved in several metabolic functions, including mitochondrial respiration and lipid metabolism suggesting bioenergetic perturbations in dopaminergic neurons (Donmez and Outeiro, 2013; Zambon et al., 2019). Additionally, α-syn interacts directly and indirectly with mitochondria and the mitochondrial recycling pathway. In fact, stable rat neurons expressing A53T, but not wild-type α-syn, showed significant accumulation of autophagic vesicles containing mitochondria (Stefanis et al., 2001). Hence, the inference is that mutant ASYN impairs mitophagy (Stefanis et al., 2001).

Mutations in the LRRK2 gene are the most frequent cause of autosomal dominant monogenic PD. The LRRK2 protein is primarily localized in membrane microdomains, multivesicular bodies, and autophagic vacuoles (Alegre-Abarrategui et al., 2009; Rideout and Stefanis, 2014), and it has been linked to autophagy and phagocytosis (Stoker and Greenland, 2018). Mutations in LRRK2, such as G2019S (kinase domain) or ROC/COR domain (R1441C, R1441H and R1441G), enhance the protein’s kinase activity, and lead to impaired vesicular trafficking, lysosomal activity and mitochondrial function in PD brains (Ryan et al., 2015). Particular mutations alter different steps of macroautophagy (Kuhlmann and Milnerwood, 2020; Madureira et al., 2020), such as autophagosome-lysosomal fusion and cargo degradation (Blauwendraat et al., 2020). Indeed, many LRRK2 mutant cell models have accumulated large autophagic vacuoles and multivesicular bodies containing incompletely degraded material (Alegre-Abarrategui et al., 2009), together with altered mTOR and AMPK activity (Ferree et al., 2012). Collectively, these studies indicate a direct relation between defects in autophagy and LRRK2 gain-of-function. In addition to effects on macroautophagy, LRRK2 mutations were also reported to affect CMA (Orenstein et al., 2013) and mitophagy (Hsieh et al., 2016; Korecka et al., 2019), so most of the clearance routes have been shown to be altered in LRRK2-PD. Furthermore, iPSCs cultures derived from patients carrying G2019S or R1441C mutations have defects in mitochondrial dynamics, as well as alterations in dinucleotide metabolism (Sanders et al., 2014) or abnormal accumulation of autophagic vacuoles (Rosenbusch and Kortholt, 2016). Similarly, animal models overexpressing LRRK2-G2019S show mitochondrial dysfunction (Rosenbusch and Kortholt, 2016). In summary, these data indicate that LRRK2 pathogenic mutations can impair autophagy, and diminish mitochondrial function and vesicular trafficking.

GBA is a glucosylceramide hydroxylase (GCase), which is important for sphingolipid degradation (Alcalay et al., 2015). GBA gene variants are towards the upper limit for risk factors, being the single greatest risk factor for PD. The initial observation of higher incidence of PD in families with Gaucher disease, a lysosomal storage disorder (LSD) caused by deficiency of the GCase, has led to the identification of heterozygous mutations in GBA as important and common risk factors for sporadic PD (Billingsley et al., 2018). Although enzymatic activity of GCase in GBA heterozygous mutants is not well known, an increased production of reactive oxygen species, and affected autophagolysosome function have been shown in fibroblasts from GBA heterozygotes with or without PD (McNeill et al., 2014; Magalhaes et al., 2016). Interestingly, all these findings in GBA mutants suggest a relation between altered lipid metabolism, bioenergetics and autophagy-lysosomal dysfunction in PD pathogenesis. For instance, α-syn accumulation has been described in the brain of patients with lysosomal disorders, as well as in several mouse models of lysosomal storage diseases. Hence, lipid accumulation could contribute to neurotoxicity (Shachar et al., 2011; Nelson et al., 2014, 2018; Smith et al., 2014).

On the other hand, mitochondrial dysfunction and autophagy defects have also been reported in Gaucher disease cellular models, such as iPSC-derived neurons from GBA-PD patients, primary post-mortem brain tissue from GBA heterozygous patients or primary hippocampal neurons from GBA L44P knock-in mouse brains. (Osellame et al., 2013; Gegg and Schapira, 2016; Schöndorf et al., 2018; Li et al., 2019; Hou et al., 2020).

## 4 Defects of Nutrient Signaling and Autophagy in ALS/FTD

### 4.1 Evidences of Poor Nutrient Utilization and Autophagy Dysregulation in ALS/FTD Patients

All over these last few decades, ALS has come to be recognised as a complex syndrome, not only due to its heterogeneous clinical display, but also to its newly discovered clinical manifestations affecting fundamental systemic processes such as metabolism and autophagy. ALS is therefore considered a systemic condition, rather than a solely neurological disease (Dupuis et al., 2011; Robberecht and Philips, 2013; Riancho et al., 2019). ALS is often related to another neurodegenerative disorder with non-motor symptoms, the frontotemporal lobar dementia (FTD) (Lau et al., 2018), as they both share common clinical and pathological phenotypes—most notably, the existence of TDP-43 positive aggregates -, and even genetic causality (Robberecht and Philips, 2013; Pang and Hu, 2021); thus, they have been suggested to be part of the same spectrum of disease (Neumann et al., 2006). In this respect, bioenergetic alteration and activation of the unfolded protein response (UPR) are also key events of FTD.

#### 4.1.1 Metabolic Alterations Present in ALS Patients

Neurodegeneration is frequently linked to, and potentially arises from a defective energetic metabolism (Dupuis et al., 2011). In the same fashion as in other neurodegenerative disorders such as Parkinson’s or Alzheimer’s disease (Pradat et al., 2010; Błaszczyk, 2020), metabolic homeostasis of ALS patients is severely altered (Tefera et al., 2021). Of note, most of the occurring abnormalities correlate with life expectancy and have a negative impact on overall pathogenic process (Dupuis et al., 2011).

One of the hallmarks of the metabolic alterations observed in ALS is the loss of the energetic balance, primarily due to a higher energetic expenditure. In other words, patients with ALS frequently exhibit a hypermetabolic state characterised by an increase of energetic expenditure, loss of body mass and depletion of energetic deposits as the disease progresses (Bouteloup et al., 2009; Dupuis et al., 2011; Zufiría et al., 2016; Vandoorne et al., 2018). Despite the uncertainty regarding the importance and causality of such dysregulation, it is worth mentioning that body weight loss is a fundamental prognostic factor for the patients, who are often associated with a lower baseline BMI, and being the disease prevalence 40% lower among obese individuals (O’Reilly et al., 2013; Perera and Turner, 2016; Vandoorne et al., 2018). In this sense, alterations in lipid homeostasis have also been described, reporting an increased LDL/HDL ratio and also higher triglyceride and cholesterol levels in ALS patients compared to controls, exhibiting a rather protective effect (Dupuis et al., 2008). Nonetheless, recently performed case-control studies on peripheral lipid profile of patients with ALS have found little or no discriminatory lipid signature (Fernández-Eulate et al., 2020; Sol et al., 2021).

Remarkably, an obesity-related condition as T2D also seems to act as a protective state for the development of ALS, delaying the disease onset up to 4 years (Mariosa et al., 2015; Tefera et al., 2021). On the other hand, type I diabetes would act as a risk factor (Jawaid et al., 2015). In accordance with this, abnormal glucose tolerance has been observed in ALS patients (Pradat et al., 2010); however, there was controversy as to whether this intolerance was inherently disease-related or due to a lack of glucose utilisation as a result of muscle atrophy, or even to an increased level of free fatty acids (Reyes et al., 1984; Perera and Turner, 2016).

Interestingly, despite the mentioned evidence of the systemic hypermetabolic state of the patients, several performed PET and autoradiography studies using glucose analogues have demonstrated that frontal and occipital cortex in the CNS contrarily exhibit a hypometabolic state, while midbrain areas would remain hypermetabolic (Pagani et al., 2014; Endo et al., 2017; Germeys et al., 2019; Tefera et al., 2021). To understand the controversy between the reported hypermetabolism at a systemic level and hypometabolism at a central level in patients, it is key to acknowledge the different nature of processes to which they refer. The systemic hypermetabolic state indicates increased oxygen consumption and is recognised as an adaptive response to satisfy prolonged increased energy demands or to cope with the inefficiency with which energy is utilized, through the mobilization of energy stores. However, the CNS hypometabolic state is ascribed to an inability of cells to take up glucose due to a state of reduced neuronal cell density, reduced blood flow and reduced glucose transporter expression (Tefera et al., 2021), while the central hypermetabolic state is rather considered a result of glia hyperactivation (Haidet-Phillips et al., 2011; Cistaro et al., 2012). Both states, systemic hypermetabolism and central hypometabolism, appear to have in common one sign: the inability with which glucose is utilized to obtain energy.

The observation of an impaired glucose uptake in motor sensory cortex of ALS patients (Pagani et al., 2014) comes in line with the defective energetic balance of such population. Besides, sporadic ALS patient-derived skin fibroblasts showed markedly lowered levels of glycolytic components (Szelechowski et al., 2018), indicating an alteration of carbohydrate metabolism. Nevertheless, high glycogen concentrations in the spinal cord of autopsied patients have been observed (Vandoorne et al., 2018), both in neurons and glia (Neumann et al., 2006). All these findings together suggest a loss of the natural coupling between blood flow and glucose metabolism in the CNS. Importantly, a widespread prefrontal glucose hypometabolism has been linked to worsened prognosis and shorter survival of ALS patients (Rajagopalan and Pioro, 2019).

It has been suggested that multiple environmental modifiers known to influence energy metabolism, affect the disease onset and/or its course (Zufiría et al., 2016). One of the most controversial activities has been physical exercise, as it is a potent modifier of muscle energy metabolism (Dupuis et al., 2011; Perera and Turner, 2016; Zufiría et al., 2016), but its exact repercussion over the disease is still unknown and many of the performed studies have come up with contradictory results. Case-control studies carried out over a population from various European countries came across the fact that not only strenuous physical exercise was related to the precipitation of the disease, but also that moderate exercise exerts neuroprotective effect (Gallo et al., 2016; Julian et al., 2021). Studies carried out among amateur and professional skiers in Sweden reported that professional athletes—but not amateurs-where related to a higher risk of developing ALS (Fang et al., 2016). Equally, another retrospective cross-sectional observational study carried out on triathletes found a drastically high representation of such athletes as ALS patients (Gotkine et al., 2014). Therefore, these two researches provide evidence suggesting that high-intensity physical exercise might exacerbate the risk of developing ALS.

As essential players in cellular metabolism, several studies have reported mitochondrial abnormalities in ALS patients (Perera and Turner, 2016; Vandoorne et al., 2018; Tefera et al., 2021). Some of the most representative ones are the presence of mitochondrial aggregates in skeletal muscle of patients (Sasaki and Iwata, 2007), and altered dynamics (Tsitkanou et al., 2016) structure, localization, volume and number in motor neurons, muscle and intra-muscular nerves (Perera and Turner, 2016). It is worth noting that mitochondrial function would also be disrupted, particularly regarding alterations in the electron transport chain, displaying reduced activity of the key enzyme complexes of the electron transport chain (Wiedemann et al., 2002; Smith et al., 2019).

Notably, motor neurons appear to be especially vulnerable to these defects in energetic homeostasis (Vandoorne et al., 2018; Ragagnin et al., 2019) due, at least in part, to their need to fulfil unusually high energetic demands to maintain resting membrane potential and propagate action potentials (Perera and Turner, 2016). Importantly, different types of motor neurons have been shown to be differentially sensitive to the disease associated damage, with the neurons innervating type IIb fibres being the most sensitive ones (Nijssen et al., 2017). Interestingly, it is this latter type which most clearly relies on glycolytic metabolism for energy (Frey et al., 2000), again suggesting an alteration in energy metabolism in the mechanism of ALS.

#### 4.1.2 Autophagy-Related Alterations in Patients With ALS

The pathology of ALS/FTD is typically accompanied by marked alterations in a key physiological process that is related to energy homeostasis: autophagy (Sala et al., 2012). Since the deletion of autophagy-related genes is sufficient to cause neuronal death, autophagy is considered an essential homeostatic process for neurons (Evans and Holzbaur, 2019).

In general, most neurodegenerative diseases, including ALS and/or FTD, are characterised by a common pathology comprising misfolded protein inclusions (Lee et al., 2015; Crippa et al., 2016; Torres et al., 2021), which eventually are responsible for degeneration or damage of nerve cells in the form of oxidative and ER stress, mitochondrial dysfunction and neuroinflamation (Ghavami et al., 2014; Corti et al., 2020; Luo et al., 2020). In the specific case of ALS, most of the mutant proteins associated to the familial inheritance of the disease exhibit aggregation-prone domains, and thus must be eliminated to prevent motor neuron toxicity (Zhang et al., 2020). Motor neurons are particularly sensitive to toxicity caused by protein misfolding, although it can also affect other cell types, such as muscle (Crippa et al., 2013). These defective proteins disrupt the ubiquitin-proteasome system, which is one of the central degradation systems for the cell, and thereby initiating a vicious cycle that culminates with further protein deposition leading to the formation of inclusions (Crippa et al., 2010).

The mentioned protein inclusions present in ALS/FTD generally consist of proteins involved in the intracellular degradation systems, various signalling systems, and in particular, TDP-43 (Neumann et al., 2006; De Marco et al., 2011). The neuropathology associated with TDP-43 mutations is characterised by ubiquitin-positive inclusions in neuronal and glial cells in the spinal cord and brain of ALS and FTD patients (Arai et al., 2006; Zhang et al., 2009). Besides, TDP-43 aggregates are characterised by the presence of phosphorylated forms of the protein, both in its full form and in fragments (Crippa et al., 2016). Ubiquitin positive TDP-43 aggregates co-localize with autophagy markers in spinal motor neurons of sporadic ALS patients (Burk and Pasterkamp, 2019).

Several studies suggest that activation of autophagy may be protective in some neurodegenerative diseases, given the potential removal of toxic protein species (Wong and Cuervo, 2010; Chen et al., 2012; Jaronen et al., 2014). Under normal conditions, a HSP protein-driven chaperone-mediated form of autophagy takes place in brain and muscle (Corti et al., 2020). In this very sense, abnormally high concentrations of the autophagy-enhancing chaperone HSPB8 have been detected in the spinal cord (Anagnostou et al., 2010), skeletal muscle (Crippa et al., 2013) and surviving motor neurons (Crippa et al., 2016) of ALS patients, as a possible strategy of the damaged cells trying to get rid of the toxic aggregates. In parallel, a number of post-mortem and experimental neuropathological studies have revealed an increase in the number of autophagosomes in motor neurons of the spinal cord of both sporadic and familial cases of ALS (Chen et al., 2012; Riancho et al., 2019).

In this sense, changes in ER morphology have been reported in patients with ALS (Zufiría et al., 2016). For instance, fragmentation of the rough ER, irregular distention of its cisternae and detachment of ribosomes have been described in degenerating anterior horn cells of post-mortem samples of patients with ALS (Oyanagi et al., 2008). Another study showed deposits of granular or amorphous material in ER lumen of sporadic ALS cases, thereby suggesting that the accumulation of misfolded proteins could be the underlying cause of stress at the ER level (Sasaki, 2010).

The cause behind ER stress in ALS/FTD and subsequent activations of UPR and autophagy is not clear yet. One of the postulated explanations suggests that ER stress could arise from the accumulation of oxidative stress (Jaronen et al., 2014). Supporting this hypothesis, analysis of blood, urine and cerebrospinal fluid samples from ALS patients has revealed an upregulation of oxidative stress markers (Riancho et al., 2019). Another possible explanation is that ER stress arises from an imbalance in calcium signalling (Grosskreutz et al., 2010; Leal and Gomes, 2015) as there is evidence according to which ALS disease has been found to be associated with low calcium content at the ER (Jaiswal and Keller, 2009; Tedeschi et al., 2019). Besides, motor neurons happen to be especially sensitive to calcium signalling dysregulation, as their intracellular Ca^2+^ levels must be tightly controlled to assure their proper function (Nijssen et al., 2017).

Beyond these postulates, the dysregulation of metabolic pathways and/or the poor utilization of energy resources, either of which are clearly present in ALS/FTD, presumably play key roles in the mechanisms behind the induction of pathological ER stress and autophagic dysregulation in neurons. As further reviewed, the fact that many of the genes that are found mutated in familial cases exert key functions in cellular energy homeostasis and recycling mechanisms provides strong evidence on this link. In this regard, AMPK is plausibly a key mediator of the activation of autophagy in response of energy scarcity in the pathological context of ALS/FTD. AMPK besides its role as metabolic sensor (Hardie et al., 2016), upon activation, AMPK turns on autophagic flux as mentioned above. In this sense, enhanced levels of activated AMPK have been observed in spinal motor neurons from patients with ALS (Lim et al., 2012a; Liu et al., 2015b), providing a compelling explanation for the increased number of autophagosomes in such neurons. However, it is not clear yet whether chronic AMPK activation as an adaptive response of affected neurons against energetic stress may produce beneficial outcomes or further precipitate the pathological crisis as it would interrupt the overall synthesis of proteins and over-activate the degradation of cellular components by the lysosomes (Figure 3).

### 4.2 Involvement of ALS/FTD Genes in Energetic and Autophagic Homeostasis

In the last decade, multiple studies have identified several disease-causative genes that overlap in both disorders. Such genetic mutations have been linked to different types of specific cytoplasmic inclusions and molecular signatures, which point to convergence, regarding cellular processes and pathomechanisms (Blokhuis et al., 2013). Although the vast majority of ALS-FTD cases have idiopathic or sporadic origin, unrevealing the genetic basis of the disease might be of great interest; and, considering that mutations in disease-causative genes have been also described in sporadic cases (Chen et al., 2013), similar pathogenic mechanisms could be present in different ALS-FTD subtypes (Vandoorne et al., 2018).

#### 4.2.1 ALS/FTD Genes and Regulation of Energy Metabolism

TARDBP, FUS, SQSMT1, VCP, TBK1, CHCHD10, C9ORF72, OPTIN, UBQLN2 and SOD1 are the most significant disease-causative genes that share these two neurodegenerative conditions. Such genes are implicated in several cellular processes, including RNA metabolism, autophagy-lysosome axis and energy metabolism *via* mitochondrial respiration, pointing out them as the central pathways involved in ALS-FTD disease spectrum.

TDP-43 and FUS-proteinopathies are characterized by the formation of cytoplasmic ubiquitinated and/or hyperphosphorilated inclusions (Neumann et al., 2006; Kwiatkowski et al., 2009; Xu et al., 2010). The translocation of both TDP-43 and FUS onto the cytoplasm implies a loss-of-function in the nucleus disrupting RNA and protein homeostasis, which may provoke a vicious cycle causing further deleterious consequences. It is described that energy homeostasis is impaired in ALS-FTD (Vandoorne et al., 2018). These two ribonucleoprotein aggregates are implicated directly on it by interfering, for example, in mitochondria functionality. Mutations in FUS, specifically P525L, lead mitochondria to fragmentation. Deng et al. (2015), observed in flies carrying mutant-FUS that its translocation occur, mediated by HSP60 (a mitochondrial chaperonin), damaging mitochondrial integrity and disrupting mitochondrial cristae (Deng et al., 2015). Not only in flies, but also in P525L-FUS expressing cells and patients, mutated FUS expression provokes reduced mitochondrial membrane potential and an increment on ROS production. Transgenic mice overexpressing WT-FUS develop a motor phenotype and display early structural abnormalities mitochondrial in the pre-synaptic motor nerve (So et al., 2018).

TDP-43 has been also related with mitochondrial dysfunction, HEK293 cells overexpressing WT or mutant TDP-43 showed reduced mitochondrial membrane potential, oxygen consumption rate, and as a consequence, low ATP levels (Wang et al., 2016). These defects are likely to be mediated by the accumulation of mutant forms of TDP-43 in mitochondria leading to impairments in complex I activity, as shown in neurons from patients with ALS (Tefera et al., 2021).

Besides its involvement in mitochondrial energy production, TDP-43 has also proven to be fundamental for fat and glycolytic metabolism (Stallings et al., 2013). The exact metabolic pathway in which it takes part remains to be clarified, but several studies have suggested that TDP-43 participates in the mechanisms of nutrient sensing and glucose uptake in motor neurons and other cells such as islet β-cells, through regulation of AMPK or adiponectin signalling, or controlling the levels of the Rab GTPase-activating protein Tbc1d1 (Ling et al., 2014; Perera and Turner, 2016; Zufiría et al., 2016). TDP-43 has also emerged as a key regulator of insulin secretion, as it exerts transcriptional control over crucial components of the insulin secretion machinery, such as UNC13A, ci-Ins2 and CaV1.2 RNAs (Araki, 2019; Stoll et al., 2020; Brown et al., 2021).

Coiled-Coil-Helix-Coiled-Coil-Helix Domain 10 (CHCHD10) is a protein with several functions involved in mitochondrial metabolism (e.g., regulation of ETC components’ synthesis) (Zhou Z.-D. et al., 2017). CHCHD10 is located in the intermembrane space, and its mutated forms’ overexpression in HeLa cells leads to fragmentation of mitochondrial network. Furthermore, patients’ muscle as well as fibroblasts showed deficient ETC activity, along with abnormalities in cristae morphology (Bannwarth et al., 2014). A recent study performed with muscle conditional KO-CHCHD10 mice, reported that these mice showed motor defects, aberrant NMJs and thereby disturbed neuromuscular transmission (Xiao et al., 2020). Interestingly, exogenous ATP contributed rescuing the NMJ defects in KO-CHCHD10 muscles. The loss of CHCHD10 function in motor neurons elicits an energy deficit that activates unique responses to nutrient stress in both the mitochondria and ER, including AMPK signaling and UPR (Straub et al., 2021).

Valosin-containing protein (VCP), an ER protein that fundamentally functions as a driver of misfolded proteins to proteosome during ER stress, is also involved in mitochondrial fitness and thereby contribute to the cellular energetic balance. In this regard, patients carrying VCP mutations leads to severe mitochondrial uncoupling, resulting in decreased ATP generation, making neuronal cells more vulnerable to energy-demanding processes (Bartolome et al., 2013).

#### 4.2.2 Role of ALS/FTD Genes in Autophagy

As mentioned above, many of the overlapping genes in ALS-FTD are involved in autophagic pathway. Interestingly, the main hallmark of this neurodegenerative condition are cytoplasmic aggregates that are not being degraded probably due to non-functional autophagic flux. Its impairment results in reduced cell survival, and thereby causes progressive degeneration.

TANK binding kinase 1 (TBK-1) is a protein kinase that belongs to the IKK-kinase family, which is involved in innate immune system. It is also a key player in autophagy, mainly regulating phosphorylation of autophagy and mitophagy adaptors (e.g., SQSTM1 and OPTN) (Pilli et al., 2012). Mutations in TBK1 has been reported as disease-causative in both ALS and FTD, or ALS-FTD continuum (Freischmidt et al., 2015). HEK293T cells transfected with mutant TBK1 compared to WT, showed diminished phosphorylation and reduced homodimerization, which are essential for TBK1 activation. These results were further confirmed using patients’ derived lymphoblastoid cell lines carrying missense mutations in TBK1, where phospho-TBK1 was found reduced, meaning that TBK1 activation is altered because of the reduced phosphorylation by self-interaction or with other kinases (de Majo et al., 2018). Using live-cell imaging to examine mitophagy dynamics after mitochondrial depolarization in HeLa cells, it has been observed that TBK1 and its downstream target OPTIN are required for engulfment of damaged mitochondria. And, specially, it has been seen that loss or chemical inhibition of TBK1, as well as expression of TBK1^E696K^, OPTIN^W478G^ and OPTIN^Q398X^, which are ALS-linked mutations, impair mitophagy, thereby accumulation of damaged mitochondria occurs (Moore and Holzbaur, 2016).

As mentioned above, Optineurin (OPTN) is a downstream target of TBK1, which is involved in several vesicular trafficking pathways. Many ALS-linked mutations in OPTN map within the ubiquitin-binding domain (UBD) (Maruyama et al., 2010), which lead to increased OPTN-immunoreactive cytoplasmic inclusions. Neuron2A cells expressing UBD-mutated OPTIN showed an accumulation of LC3-II-positive inclusion bodies by decreasing autophagy-mediated degradation (Shen et al., 2015). ALS-associated OPTN mutants expression in NSC-34 motor neuron-like cell line, showed disrupted interaction between OPTN and myosin VI, resulting in interrupted protein trafficking, as well as endoplasmic reticulum stress and Golgi fragmentation. Moreover, implication of OPTN in lysosome trafficking during autophagy in association with myosin VI has been reported, since ALS-mutant expression as well as knockdown of OPTN lead to blockage of lysosome-autophagosome fusion, hence accumulating autophagosomes in neuronal cells (Sundaramoorthy et al., 2015).

Sequestosome-1(SQSTM1) encodes p62, a multifunctional protein that has an essential role in selective autophagy, as well as regulating mTOR-signalling pathway. Some of ALS-FTD associated mutations in SQSTM1 map to the LC3-interactin region, whereby lipid-anchored form of LC3 is bound, allowing the phagophore to evolve in autophagosomes (Stamatakou et al., 2020). Goode et al. (2016) demonstrated that the ALS-associated L341V mutation of SQSMT1 affects on the recognition of LC3, reducing binding affinity. These results that were obtained from experiments done in motor neuron-like cells with the L341V mutant SQSMT1, showed more difficulties for incorporation into autophagic vesicles (Goode et al., 2016). In addition to this loss-of-function of the protein, many papers have reported that there is also a gain-of-toxicity which is manifested through p62-positive cytoplasmic inclusion, and not only in neurons, but also in glial cells (Arai et al., 2003). Recent studies have revealed that ALS-FTD linked mutations in SQSTM1 reduce SQSTM1 phosphorylation, which is necessary for activation of selective autophagy, as well as impair KEAP1-SQSMT1 interaction (essential for antioxidant response activation), leading to increased TDP-43 associated aggregates (Deng et al., 2020).

Ubiquilin 2 (UBQLN2) is a chaperon protein that transports ubiquitinated cargoes to be degraded by ubiquitin-proteasome system (Zhang et al., 2014), playing a key role in protein quality control network (Shahheydari et al., 2017). It is also necessary for autophagosome maturation since its reduction leads to decreased autophagosome formation (Rothenberg et al., 2010). Interestingly, UBQLN2 has been related with mTOR, an essential negative regulator of macroautophagy, highlighting its involvement on the autophagic flux (Jung et al., 2010; Şentürk et al., 2019). Mutations in UBQLN2, which are linked to chromosome-X, cause dominantly inherited ALS-FTD (Deng et al., 2011). Recent studies have shown that flies with ALS associated UBQLN2 mutation display defective autophagic flux due to the impaired interaction with the v-ATPase proton pump, which is responsible for lysosome acidification. Therefore, loss of ubqln2 provokes lysosome alkalization, impairing the degradation process; which can be ameliorated in flies by acid nanoparticles for lysosome re-acidification (Şentürk et al., 2019). Consistent with this data, human HeLa cells carrying UBQLN2P497S mutation, show disturbances in proteostasis through interferences in autophagy pathway, as well as in HeLa UBQLN2-KO cell line, in which autophagosome acidification is impeded. Nevertheless, exogenous WT UBQLN2 expression is enough to restore autophagosome acidification almost as normal HeLa cells levels (Wu et al., 2021).

Valosin-containing protein (VCP) in addition to its role in mitochondrial respiration, it is also involved in ubiquitin-containing autophagosomes maturation (Johnson et al., 2010; Tresse et al., 2010). Interestingly, Tresse et al. (2010) demonstrated that VCP is essential for autophagosome maturation at a late stage after acidification; since MEFs expressing tagged-LC3 and mutated-VCP have shown accumulation of immature autophagic vesicles, some of which are acidified and abnormally large and. Other studies have proven the implication of VCP in autophagy by knocking it down, and as a result accumulation of autophagosomes have occur (Ju et al., 2009).

An expansion of GGGGCC hexanucleotide within the C9ORF72 gene was identified as an ALS-FTD causative mutation (DeJesus-Hernandez et al., 2011; Renton et al., 2011). When it was discovered, its biological function persisted unknown for many years. Nowadays, it has been included as an important player in the function and homeostasis of the lysosome (Amick and Ferguson, 2017). In addition, both human and mouse cell models lacking/expressing mutant- C9orf72 have shown abnormal lysosomes, and together with that, defects in autophagy and lysosomal degradation (Root et al., 2021). This may reinforce the aberrant upregulation of mTORC1 seen in c9orf72 and smcr8 double-knockout (dKO) mice, resulting in mTOR signalling overactivation, due to the disruption of autolysosome acidification. (Shao et al., 2020). In Drosophila studies, it has been shown that the repetition of the GGGGCC hexanucleotide impairs the nuclear import of Mitf/TFEB, a transcriptional regulator of autophagolysosomal function, leading to autophagy disruption, accumulation of lysosome-like organelles and proteostasis prior to neurodegeneration (Cunningham et al., 2020).

As discussed above, AMPK is plausibly a key protein mediating a link between the abnormalities of energy producing pathways and autophagy, which are central to the pathological continuum of ALS/FTD; and as such, dysfunction of various causative genes is associated with impaired AMPK signalling. For instance, pathological expression of TDP-43 or mutant SOD1 and CHCHD10 elicits intense activation of AMPK in mice spinal cords and motor neurons (Lim et al., 2012b; Liu et al., 2021; Straub et al., 2021). Importantly, activation of AMPK induces some of the ALS/FTD proteins to mislocalize or change its functional status, as observed for TDP-43 and TBK1 (Zhao et al., 2018; Liu et al., 2021), suggesting that AMPK activation is not a mere epiphenomenon but establishes a bidirectional crosstalk with ALS/FTD pathology. In this sense, suppression of AMPK activity exerted consistent improvements of motor functions in TDP-43 overexpressing mice (Liu et al., 2015a).

### 4.3 Abnormal Lysosome Function in FTD-GRN as Example of Defective Integration Between Nutrient Sensing and Nutrient Recycling in Neurodegeneration

As explained above, the lysosome is a chief metabolic hub that controls the metabolic status of the cell in response to the environmental changes. Failure of lysosomes has been implicated in the pathogenesis of neurodegeneration, metabolic disease and cancer (Ballabio and Bonifacino, 2020).

The lysosome performs very relevant functions in the central nervous system for the correct maintenance of the cell homeostasis. The central nervous system requires higher rates of macromolecules synthesis and organelles for the proper neuronal plasticity, and autophagy for the degradation of damaged proteins and organelles, which cannot otherwise reduce concentration by cell division (Root et al., 2021). In fact, mutations in genes linked to the lysosome produce complex diseases that are commonly accompanied by neurological syndromes. An extreme example of this is the haploinsufficiency of the lysosomal protein progranulin (PGRN), which causes neurodegeneration of the frontotemporal lobe with TDP-43 pathology (FTD) (Kao et al., 2017).

More than 50 different pathogenic granulin (GRN) mutations have been identified in patients with FTD (Baker et al., 2006; Cruts et al., 2006). Most of the mutations lead to a sequence frameshift and premature stop codons. This results in mutant mRNA transcripts, which undergo nonsense-mediated mRNA decay which leads to a reduction of the precursor protein, PGRN, and its proteolytic products, granulins (Baker et al., 2006; Cruts et al., 2006). While heterozygous mutations of GRN cause an adult age onset FTD with ubiquitinated TDP-43 inclusions and behavioural, agrammatism and motor speech deficits (bvFTD, nfvPPA) (Ferrari et al., 2019), homozygous GRN mutations produce a different and more aggressive juvenile onset of a LSD known as neural ceroid lipofuscinosis (NCL), characterized by abnormal lipopigment deposition in dysfunctional lysosomes (Mole et al., 2019) (Figure 3).

PGRN has been implicated in many physiological processes ranging from cell-cycle progression, cell migration, neurotrophic signalling, wound repair, modulation of inflammation, tumorigenesis, and metabolic fitness (Van Damme et al., 2008; Bateman and Bennett, 2009), acting upstream these processes by keeping lysosomes healthy (Rideout and Stefanis, 2014; Lui et al., 2016; Paushter et al., 2018). PGRN can play a direct or indirect role on the lysosome acidification and enzymatic activity of lysosomal enzymes (Tanaka et al., 2017). Although a portion of PGRN is secreted to the extracellular space, the majority of intracellular PGRN localizes within lysosomes. It has been reported that PGRN and/or granulins can regulate the enzymatic activity of (GBA) (Zhou et al., 2019; Valdez et al., 2020), β-hexosaminidase A (HexA) (Chen et al., 2018) and CTSD, lysosomal enzymes that are important for proper lysosomal activity (Beel et al., 2017; Valdez et al., 2017). On top of that, a relevant evidence linking the role of PGRN with the lysosome is that GRN expression is regulated by the transcription factor TFEB (Belcastro et al., 2011), responsible for the activation of the CLEAR, which regulates the expression of genes involved in autophagy and lysosomal pathway (Settembre et al., 2011).

PGRN was first linked with a lysosomal function when homozygous GRN mutations were discovered to cause NCL (Smith et al., 2012). Importantly, the accumulation of storage material and multillamelar bodies was discovered in post-mortem cortical brain tissue and cells from FTD patients with heterozygous GRN mutations (Ward et al., 2017). Accordingly, GRN-deficient mice models display NCL-like phenotypes (Ahmed et al., 2010; Petkau et al., 2012; Wils et al., 2012; Filiano et al., 2013; Zhou, 2017) together with lysosomal defects in brains (Wils et al., 2012; Zhou, 2017). Collectively, these evidences raise lysosomal dysfunction as a central neurodegenerative process that is caused by PGRN insufficiency and linked to TDP-43 pathology (Ward et al., 2017).

The lysosome is a control hub for cellular growth and survival (Settembre et al., 2013; Mony et al., 2016; Lawrence and Zoncu, 2019). In line with this, PGRN was first related to metabolic disease when it was seen that the serum PGRN levels were increased 1.4-fold times in individuals with visceral obesity and T2D (Youn et al., 2009). Not only increased PGRN levels in blood correlated positively with body mass index (BMI), fat mass, fasting glucose and insulin levels, and insulin resistance (Hossein-Nezhad et al., 2012), but also mediated TNF-a-induced insulin resistance through the regulation of IL-6 expression in 3T3-L1 adipocytes. Matsubara et al. proved that PGRN promotes IL-6 expression preventing the insulin induced phosphorylation of IRS-1 and AKT, suppressing insulin-stimulated glucose uptake and causing TNF-α induced insulin resistance (Matsubara et al., 2012). Chronic inflammation is a well-known consequence of obesity in T2D (Romeo et al., 2012). These patients have increased secretion of cytokines and chemokines as TNF-α and IL-6 and the recruitment and activation of innate immune cells (Taube et al., 2012). The previously mentioned evidence also supports that PGRN acts as a adipokine increasing IL-6 levels and being regulated by TNF-α treatment (Okura et al., 2010). This evidence suggests that PGRN could be regulated by the increased inflammatory cytokines and promote insulin resistance.

Although the role of PGRN is mostly related to the lysosome the primary role that was attributed to the granulin peptides was the modulation of cell growth, so initially it was considered a growth factor (He and Bateman, 2003). The treatment with recombinant PGRN showed stimulation of proliferation of two epithelial cell lines (Daniel et al., 2000). In relation to this PGRN is the only growth factor that is able to induce cell-cycle in IGF-I-receptor negative mouse embryo fibroblasts that are not able to complete the cell cycle (Zanocco-Marani et al., 1999). Moreover, PGRN is able to stimulate endothelial cell migration and vessel growth *in vitro* and *in vivo* (He et al., 2003; Toh et al., 2013). PGRN is expressed in proliferating blood vessels and induces the expression of vascular endothelial growth factor (VEGF) a key mediator of angiogenesis (Gonzalez et al., 2003; Tangkeangsirisin and Serrero, 2004). It has been shown that PGRN is able to bind to the receptor Tyrosine Kinase, EPH receptor A2 (EphA2) and activate MAPK and AKT signalling pathways (Zanocco-Marani et al., 1999; Neill et al., 2016). In relation with the mitogenic and angiogenic functions, PGRN has been associated to several cancers, generally with a direct correlation between the PGRN levels and cancer malignancy (Tangkeangsirisin et al., 2004; Abrhale et al., 2011; Serrero et al., 2012; Edelman et al., 2014). In the other hand, reducing PGRN levels by direct antibody treatment against PGRN have a tumour suppressor effect (Ho et al., 2008).

PGRN is a well-established modulator of immune function a very relevant physiological process that takes part in cancer, metabolic disease and neurodegeneration (Toh et al., 2011; Cenik et al., 2012; Jian et al., 2013; Tanaka et al., 2013; Kao et al., 2017). Microglia, the resident immune cells in the brain, are the cells with the highest expression levels of PGRN, being especially elevated in those which have become reactive (Naphade et al., 2010; Zhou X. et al., 2017). It has been shown that PGRN can regulate multiple microglial function, activation, migration, phagocytosis and synapse pruning among others (Van Damme et al., 2008; Yin et al., 2010; Pickford et al., 2011; Martens et al., 2012; Lui et al., 2016). The PGRNs regulation of microglial activation is a complex regulation, where PGRN and GRN peptides have opposite inflammatory functions, with PGRN generally being anti-inflammatory and granulin peptides pro-inflammatory (Zhu et al., 2002; Kojima et al., 2009). Considering the role of PGRN as a growth factor in the periphery and its role in neurodegeneration, it is not surprising that PGRN and GRN E can function as neurotrophic factors. PGRN and GRN E promote neuron survival and neurite outgrowth *in vitro* and *in vivo* (Van Damme et al., 2008; Ryan et al., 2009; Gass et al., 2012; De Muynck et al., 2013). Whereas small-interfering RNA (si-RNA) mediated knock down of PGRN in primary rat hippocampal neurons reduces neurite arborisation (Tapia et al., 2011). Since PGRN expression is very low in the developing brain and is a protein that is secreted in an activity-dependent manner in the synapses and regulates microglia activation, its role may be more relevant in the context of neurite plasticity in the adult brain (Tapia et al., 2011; Petkau et al., 2012; Petoukhov et al., 2013).

In summary, PGRN has emerged as an essential protein for the fitness of lysosomes to integrate nutrient sensing with cellular recycling and anabolic processes to respond to energy demands. The function of PGRN in lysosomes provides further compelling explanation for the particular vulnerability of ALS/FTD degenerating neurons to the dismantlement of the fine-tuned crosstalk between energy imbalance and cellular recycling through the ALP.

## 5 Conclusion and Perspectives

Dysregulation of the energy-producing pathways due to dysfunctional mitochondria or altered glycolysis, as well as defects in the autophagic process are common pathological features of several neurodegenerative diseases, including PD, FTD, ALS and AD. The bulk evidence derived from experimental studies highlights a pathogenic loop in which metabolic perturbations and dysregulated energy-sensing pathways cause a cellular energy crisis. In such scenario of energy scarcity, the main cellular process that responds to this challenge, autophagy, is also altered, further aggravating the energy crisis due to the inability to recycle damaged mitochondria and to provide micronutrients for the biosynthesis of new molecules. Indeed, the fact that many of the genes found mutated in PD, FTD, ALS, and to a lesser extent in AD, share common functions in the regulation of mitochondrial metabolism, autophagic flux and lysosome function provides further compelling evidence for this pathogenic loop.

However, why the different diseases affect distinct brain areas and neuronal populations remains elusive. It is likely that the participation of other pathogenic mechanisms could make some neuron subtypes more vulnerable than others. In any case, the crosstalk between energy metabolism and autophagy regulation appears to be a pillar in the pathological cascade of the different neurodegenerative diseases, and therefore treatments aimed at alleviating such loop might be beneficial for either condition.
